# Comparison of antegrade robotic assisted VS laparoscopic inguinal lymphadenectomy for penile cancer

**DOI:** 10.1186/s12893-023-01935-6

**Published:** 2023-03-13

**Authors:** Mengjun Yang, Zhicheng Liu, Qi Tan, Xiaofei Hu, Yang Liu, Ling Wei, Chunyan Deng, Shikai Zhou, Nengrui Yang, Guangjie Duan, Yiming Zheng, Xuemei Li, Zhiwen Chen, Zhansong Zhou, Ji Zheng

**Affiliations:** 1 Department of Urology, Urologic Surgery Center, Xinqiao Hospital, Third Military Medical University (Army Medical University), Chongqing, 400037 China; 2 Department of Urology, Urological Surgery Research Institute, Southwest Hospital, Third Military Medical University (Army Medical University), Gao Tanyan R`d. 30, Chongqing, 400038 China; 3 Department of Radiology, Southwest Hospital, Third Military Medical University (Army Medical University), Chongqing, China; 4 Institute of Pathology and Southwest Cancer Center, Southwest Hospital, Third Military Medical University (Army Medical University), Chongqing, China

**Keywords:** Robotic-assisted surgery, Laparoscopic surgery, Penile cancer, Antegrade, Inguinal lymphadenectomy

## Abstract

**Background:**

Minimally invasive modifications of inguinal lymphadenectomy (IL), including laparoscopic IL (LIL) and robotic-assisted IL (RAIL), have been utilized for penile cancer. Comparative study is necessary to guide the decision about which minimally invasive technique to select for IL. Therefore we compared RAIL with LIL performed via an antegrade approach in terms of perioperative outcomes.

**Methods:**

We conducted a retrospective study of 43 patients who underwent RAIL (n = 20) or LIL (n = 23) for penile cancer from 2016 to 2020. The key surgical procedures and techniques are described. Complications were graded by the Clavien-Dindo classification, and operative time, estimated blood loss (EBL), lymph nodal yield, nodal positivity, postoperative drain duration, and disease recurrence during follow-up were assessed. Categorical variables were compared using chi-squared whereas continuous variables were compared by t-tests.

**Results:**

The operative time for RAIL was significantly shorter than that of LIL (median 83 vs 95 min). Significantly less blood loss was reported with RAIL than with LIL (median 10 vs 35 ml). Lymph node yield, pathological positive nodes, the hospital stay, postoperative drain duration, postoperative complications and recurrence were similar for RAIL and LIL.

**Conclusions:**

For patients with penile cancer, perioperative outcomes of RAIL and LIL were similar, but there was less blood loss, a shorter operative time for robotic cases.

**Supplementary Information:**

The online version contains supplementary material available at 10.1186/s12893-023-01935-6.

## Introduction

Penile cancer is a rare form of urological cancer, with an incidence from 0.4 to 0.6% in the United States to up to 10% in developing nations of Asia [1]. The most important prognostic factor for survival in patients with penile cancer is the extent of lymph node metastasis, which predicts not only survival but also morbidity because of the sequelae of inguinal lymphadenectomy (IL) [2–4].

Open IL (OIL) has been the gold standard for surgical management of the groin, but studies have described morbidity rates ranging from 50 to 90% [4]. In order to decrease the morbidity related to open IL, researchers introduce dynamic sentinel node biopsy and improved dissections into the treatment. However, dynamic sentinel node biopsy can not solve the morbidity problem of patients requiring IL. Improved dissections have also shown benefit in moderated decreasing complication rates, but may increase the risk of false-negative sampling and late groin recurrence. Therefore, there is an increasingly urgent need for therapies that can both control tumors and reduce the complications of IL. Recently, these efforts have centered on the development of minimally invasive modifications of IL, including laparoscopic IL (LIL) and robotic-assisted IL (RAIL), which have been utilized to improve the surgical outcomes of IL [5].

In 2006, LIL was first reported for the treatment of penile cancer [3]. Since then, a series of studies have shown similar oncological outcomes, faster postoperative recovery, fewer postoperative complications and better cosmetic effects for LIL than OIL [6–12]. However, the usage of LIL is limited by ergonomics and the steep learning curves. This led to the introduction of robotic surgery in 2009 to address the limitations of LIL [13]. The robotic-assisted platform can offer advantages including better ergonomics, increased magnification, a superior view, 3-dimensional clarity, better instrumentation and dexterity optimization while preserving the oncologic outcomes of IL [14–24].

However, it should be taken into consideration that robotic platforms can increase the cost for patients and health care systems, especially in developing countries [25]. Furthermore, to date, it is unclear whether the theoretical advantages of robotic surgery actually translate into clinical benefits [19]. Therefore, a direct comparative study is necessary to guide the decision about which minimally invasive technique to select for IL.

In this study, we compared the clinical outcomes of RAIL and LIL performed via the antegrade approach, and there was no need in this approach to re-position the robotic platform, unlike the retrograde [14–22, 24, 25] and lateral approaches [11, 12], and pelvic lymphadenectomy could be performed simultaneously using the original trocar position, if necessary [23].

## Methods

### Patients

We retrospectively analyzed the records of 43 patients who underwent inguinal lymphadenectomy surgery via an antegrade approach for penile cancer from March 2016 to August 2020 at SOUTHWEST Hospital. All of the patients diagnosed with penile cancer have undergone RAIL or LIL surgery according to their choice with authorization. The collected cases were respectively divided into two groups: the LIL group (n = 23) and the RAIL group (n = 20), based on the surgical method they previously experienced. The elaborated surgical procedures of subjects in the RAIL and LIL groups were shown in the section of “Surgical procedure” and “Supplementary Material”. All participants provided informed consent. The study was approved by the Institutional Ethics Committee of SOUTHWEST Hospital (KY2021085) and was conducted in accordance with the national guidelines and the Declaration of Helsinki. The technique and surgical steps are described below.

### Surgical procedure

#### RAIL and LIL

The patient was placed in the supine position on a split-leg table, and the bedside robotic arm of the da Vinci robot was pushed between the legs of the patient. The dissected lymph nodes were placed in a specimen bag and removed via the camera trocar incision for frozen section and pathological examination. Consequently, hemostasis was performed thoroughly on the wound, a negative-pressure drainage tube was placed via the assistant trocar, and the skin incisions were closed. For patients who had positive pathological results, pelvic lymphadenectomy was then performed simultaneously using the original skin incision with the patient’s position and position of the robotic arm not shifting as previously described [23].

The surgical procedure of LIL is identical to RAIL. Detailed surgical procedures are provided in Additional file 1: Figure S1–S3.

#### Postoperative care

After surgery, the groin area was bandaged with elastic. Patients achieved pain control with oral medications and were asked to follow a low-fat high-protein diet. The drainage tube was left in place until the output was less than 30 mL/day after ambulation.

### Statistical analysis

The statistical software package SPSS 20.0 (SPSS Inc., Chicago, IL, USA) was applied for all analyses. Continuous data are presented as the median and interquatile rang (IQR), and categorical variables are presented as the number and proportion. Categorical variables were compared using chi-squared whereas continuous variables were compared by t-tests. All statistical analyses were performed with 2-tailed tests, and P < 0.05 was considered statistically significant.

## Results

A total of 43 patients were included in the study, among which 20 patients underwent RAIL and 23 patients underwent LIL. There were no intraoperative complications or conversion to open surgery. The two groups were comparable in age, body mass index, smoking status, presence of diabetes mellitus, pathological tumor stage, and clinical and pathological nodal stage (Table 1).

**Table 1:** Demographic data for patients included in this study

	Robotic	Laparoscopic	P
n	20	23	
Age, year, median (IQR)	56 (48–65)	53 (49–65)	0.517
BMI, median (IQR)	25.0 (20.9–27.1)	23.4 (21.5–27.2)	0.703
Smoking, no. (%)	13 (65.0)	19 (82.6)	0.295
Diabetes mellitus, no. (%)	0 (0.0)	2 (8.7)	0.491
pT stage pT1/ pT2/ pT3	12/8/0	14/9/0	0.954
cN stage cN0/cN1/cN2/cN3	10/3/7/0	10/1/12/0	0.791

For RAIL, the median operative time per limb was 97 min per side, IQR ranging from 89 to 108 min, which was not significantly different from the LIL group (95 (90–115)) (P = 0.204) (Table 2). However, when the robot docking time was excluded, the operative time per limb was significantly lower in the RAIL group (median 83 vs 95 min, p < 0.001, Table 2). EBL was significantly higher in the LIL group than in the RAIL group (median 10 vs 35 ml, p < 0.001) (Table 2). Saphenous vein sparing and bilateral dissection were performed in all cases in both groups. Lymph node yield and pathological positive nodes were similar in both groups. The hospital stay (p = 0.081) and the number of days requiring drains to remain in situ (p = 0.522) was comparable in the two groups.

**Table 2:** Comparison of intraoperative and postoperative outcomes between RAIL and LIL

	RAIL	LIL	P
Operative time /limb (min)	97 (89–108)	95 (90–115)	0.204
Operative time /limb (min) (console time)	83 (78–93)		< 0.001
Estimated blood loss (ml)	10 (6–20)	35 (25–50)	< 0.001
Saphenous vein sparing, no. (%)	20 (100)	23 (100)	
Bilateral dissection, no. (%)	20 (100)	23 (100)	
Lymph node yield, median (IQR)	19 (16–25)	18 (12–21)	0.307
Pathological positive nodes, median (IQR)	3 (1–5)	3 (1–6)	0.543
pN stage pN0/pN1/pN2/pN3	15/0/4/1	16/1/5/1	1.000
Length of hospital stay, median (IQR)	3(2–4)	5 (4–7)	0.081
Duration of drainage, median (IQR)	15 (11–18)	15 (10–21)	0.522
*Complications*			0.447
Seroma, no. (%)	4(20)	2 (9)	
Lymphedema, no. (%)	2 (10)	3 (13)	
Deep venous thrombosis, no. (%)	0 (0)	1 (4)	
Cellulitis, no. (%)	0 (0)	2 (9)	
Clavien classification, no. (%)			0.762
I, n (%)	4(25)	5 (22)	
II, n (%)	0 (0)	1 (4)	
IIIa, n (%)	1 (5)	2 (9)	0.635
*Follow up*			0.401
Duration of Follow up, mo, median (IQR)	14 (4–16)	16 (14–18)	0.078
No recurrence	18	19	
Recurrence	2	2	
Loss to follow-up	0	2	

For the RAIL group, the overall postoperative complication rate was 30%, which included 20% seroma and 10% lymphedema. In the LIL group, 35% of dissections experienced postoperative complications, which included 9% seroma, 13% lymphedema, 4% deep venous thrombosis and 9% cellulitis. There was no statistically significant difference in the rate of short-term and long-term postoperative complications between the two groups.

In the RAIL group, 20 patients were followed up for 12–24 months with a median follow-up time of 14 months. No patient was lost to follow-up and two patient developed recurrence post-surgery. The recurrence-free survival rate was 90%. In the LIL group, 23 patients were followed up for 12–30 months with a median time of 16 months. Among them, 2 patients were lost to follow-up. Two patients suffered a tumor recurrence, and 1 patient died of metastasis. The remaining 19 patients had no recurrence or metastasis. The recurrence-free survival rate was 85%. (p = 0.401) (Table 2).

## Discussion

To the best of our knowledge, there are very few study that compared the clinical outcomes of RAIL and LIL performed via an antegrade approach. In terms of operative time, while some studies have reported no statistically significant difference between minimally invasive and open IL [26, 27], most papers reported a longer operative time for minimally invasive surgeries. The operative time for RAIL when excluding reports combining inguinal lymphadenectomy with other procedures (e.g., vulvectomy, pelvic lymph node dissection) was quite variable, ranging from 45 to 279 min [5, 17, 18, 24]. Similarly, various studies reported very heterogeneous results for the operative time of LIL, which ranged between 90 and 240 min [7–12]. Consistent with Russell CM et al. [19], the operative times for RAIL and LIL in our cohort were comparable (median 97 vs 95 min/limb). However, the console time for RAIL was significantly shorter than the operative time for LIL, which indicated a faster dissection after the robot was docked in place [20, 27] and the use of a robotic platform resulted in meaningful changes in procedure time.

EBL for RAIL in the studies reviewed ranged from 10 to 200 ml [14, 20]. Datas come from the research of Ji shown comparable blood loss in 10 RAIL and 11 LIL (RAIL 15 ml vs LIL 16 ml p = 0.521). Russell et al. reported comparable blood loss per groin in 27 RAIL and 7 LIL groin dissections (50 ml [range 15–50] vs. 50 ml [range 37.5–75]) [19]. Among nine RAIL groin dissections, Yu et al. estimated the median blood loss to be less than 10 ml per groin [23]. A study by Nayak et al. [11] indicated that EBL was significantly reduced with L-LIL (lateral LIL) compared to OIL, which was lower than the blood loss reported by Wang et al., who used central LIL [26]. These findings suggest that blood loss during RAIL seems to be comparable to that of LIL and not worse than that of OIL. In this study, compared with LIL, RAIL led to significantly lower blood loss, which was possible due to better visibility and enhanced flexibility, clarity, and accuracy in avoiding blood vessels in the surgical field provided by the robotic platform and was consistent with previous comparative studies of prostate, colorectal, endometrial and thyroid cancer [25].

The length of hospital stay was significantly shorter in minimally invasive IL studies than in OIL studies, although randomized controlled data are lacking [21, 26, 27]. The reported length of hospital stay after RAIL ranges from 0 to 7 days, with most authors reporting an average of 1–2 days [21, 22]. For LIL, the length of hospital stay is more variable and ranged from 1 to 62 days in previous studies [5, 10–12, 25]. According to the comparative study of RAIL and LIL reported by Russell et al. [19], the median length of stay was 1 day for both LIL and RAIL patients. In this study, RAIL was associated with a decreased hospital length of stay compared with LIL, which may be the combined result of a shorter operative time, lower blood loss, smaller subcutaneous workspace and decreased CO2 pressure of the workspace (8 mmHg) in our robotic cases.

There are still no universally accepted recommendations regarding postoperative drain management. Drains are usually kept for weeks and removed when the output is < 30–50 ml over 24 h after ambulation [25]. The median time of drainage removal reported by different researchers varied [11, 19, 20, 23] in minimally invasive surgeries. The drain durations of RAIL and LIL are extremely heterogeneous, ranging 24.5–48.5 days [19], 7–65 days [24], 7 to 72 days [5] and 5–28 days [8]. Consistent with Russell et al. [19], who reported comparable median times to drain removal days for LIL (42.5 days) and RAIL (36.0 days), the time to drain removal days was comparable in the RAIL and LIL groups in the current study.

In terms of lymph node yield, the majority of the current data suggests that minimally invasive techniques have similar lymph node yields compared with non-minimally invasive surgery [25]. Singh et al. reported no significant difference in the median number of lymph nodes when comparing open IL and RAIL in their cohort of 51 patients (12.5 vs. 13, p = 0.44) [20]. In contrast, Nayak et al. reported that the mean nodal yield and nodal positivity were significantly better in the L-LIL group than in the OIL group [11], and LIL coincides with a higher mean nodal yield than open surgery in other studies [3]. In regard to direct comparisons between LIL and RAIL, Ji et al. shown lymph nodes of RAIL vs LIL (22.2 ± 4.5 vs 15.4 ± 3.1 p < 0.01) [28]. While, in the research of Russell CM, the median number of lymph nodes from LIL was 10 (range 7.5–12) and 8 from RAIL (range 6.0–12), respectively, and this difference was not statistically significant (p = 0.84) [19]. Interestingly, our results are consistent with Russell et al. [19]. Our results show that a comparable level of lymph node yield and nodal positivity was achieved by the LIL and RAIL approaches. Most surgical oncologists use the number of lymph nodes obtained as a measure of groin dissection quality. A recent multicenter study demonstrated that 90% of ILs retrieved at least six nodes and suggested this number as the standard [29]. In this study, the median lymph node yield was 19 (IQR 16–25) in RAIL and 18 (IQR 12–21) in LIL, which confirms the oncologic adequacy of dissection.

Postoperative complication rates appear to benefit from minimally invasive approaches compared to OIL [3, 20, 21, 23, 25–27]. When comparing LIL to open IL, there was a significant decrease in wound complications (0% vs 50%) as well as a trend toward lower overall complication rates (20% vs. 70%) [25], concordant with findings reported by other investigators [16, 17]. Similarly, Singh et al. [20] reported lower complication rates with RAIL than with open IL (2% vs 17%), consistent with Yu et al. [23], who reported that RAIL had fewer postoperative wound complications. According to Russell et al. [19], lower complication rates with the RAIL approach (11% vs 43%) may result from a significantly increased rate of successful saphenous vein preservation when compared with the LIL procedure (100% vs 57%). In this study, saphenous vein sparing was performed in all cases in both groups, and the overall complication rate in RAIL (30%) was comparable to that in LIL (35%) and other LIL series (18%-41%) [3, 25–27].

Our study had several limitations. First, our patient selection was not randomized and the study was retrospective. Second, it may also be of concern that results from a single surgeon’s experience in the same hospital might not be easily reproduced in a different setting. Third, given the limited sample size and length of follow-up in our study, future research should focus on conducting large series with long-term follow-up, and randomized, prospective studies are warranted.

## Conclusions

In conclusion, this retrospective comparative study indicates that perioperative outcomes are similar for RAIL and LIL, but with less blood loss and a shorter operative time for robotic cases. Future prospective multi-institutional trials are required to more accurately determine which approach is advantageous in terms of perioperative and oncologic outcomes and to investigate their associated costs.

## Supplementary Information

**Additional file 1.** Specific surgical procedures for robot-assisted/laparoscopic inguinal lymphadenectomy.

## Data Availability

The datasets of the current study are available from the corresponding author upon reasonable request.

## Abbreviations

IL: Inguinal lymphadenectomy
OIL: Open inguinal lymphadenectomy
LIL: Laparoscopic inguinal lymphadenectomy
RAIL: Robotic-assisted inguinal lymphadenectomy
EBL: Estimated blood loss
