# Supplementary material for: Comparison of antegrade robotic assisted VS laparoscopic inguinal lymphadenectomy for penile cancer
Source: BMC Surg. 2023 Mar 13;23:55. doi: 10.1186/s12893-023-01935-6 (PMC10012593; doi:10.1186/s12893-023-01935-6)
Supplement: Supplementary file 1 — Additional file 1. Specific surgical procedures for robot-assisted/laparoscopic inguinal lymphadenectomy. [file 12893_2023_1935_MOESM1_ESM.docx]

**SUPPLEMENTARY MATERIALS**

**Surgical procedure**

**RAIL**

The patient was placed in the supine position on a split-leg table, and the bedside robotic arm of the da Vinci robot was pushed between the legs of the patient (Supplementary Figure 1). A 2 cm longitudinal incision was made at the lower margin of the umbilicus to create a workspace beneath Scarpa’s fascia. A 12 mm trocar was placed via the incision to be used as a lens hole. The workspace was expanded with CO_2_ insufflation at a pressure of 12 mmHg.

For the right IL, a metal trocar of arm-2 was placed 8-10 cm inferior to the umbilical cord at the medioventral line, and another metal trocar for arm-1 was then placed 8-10 cm lateral to the umbilical cord. Subsequently, a 5 mm trocar was placed 2 cm superolateral to the anterior superior spine as an assistant port (Supplementary Figure 1A). For the left IL, the lens hole was unchanged, the original trocar for arm-2 was used as the trocar of arm-1, and another trocar was then placed 8-10 cm lateral to the umbilical for arm-2. (Supplementary Figure 1).

The boundaries of the dissection extend from the inguinal ligament superiorly, the sartorius muscle laterally, and the adductor longus muscle medially. First, dissociation was performed from the surface of the external oblique aponeurosis to the inguinal ligament, and then the spermatic cord was identified (Supplementary Figure 2). Second, for deep plane dissection, the cribriform fascia was cut open, and the femoral vein was dissected inferomedially to enable resection of the deep inguinal nodes[16]. Then, the femoral artery and vein were exposed to let the surface skeletonize. The femoral vein was dissociated downward to expose the great saphenous vein, which was spared in all cases, and the small branches of the femoral artery and vein may be clipped and divided (Supplementary Figures 2 ). After deep plane dissection, superficial plane dissection was performed under Scarpa’s fascia, and the packet was dissected away at its superolateral and superomedial limits. This maneuver was continued inferiorly to the apex of the femoral triangle (Supplementary Figure 3).

The dissected lymph nodes were placed in a specimen bag and removed via the camera trocar incision for frozen section and pathological examination. Consequently, hemostasis was performed thoroughly on the wound, a negative-pressure drainage tube was placed via the assistant trocar, and the skin incisions were closed.

For the left-side procedure, the robot maintained the same position, and the trocar position was the same as that on the right side but in mirror mode (Supplementary Figure 1).

For patients who had positive pathological results, pelvic lymphadenectomy was then performed simultaneously using the original skin incision with the patient’s position and position of the robotic arm not shifting as previously described [23].

**LIL**

The surgical procedure of LIL is identical to RAIL.

**Supplementary Figure and legends**

**
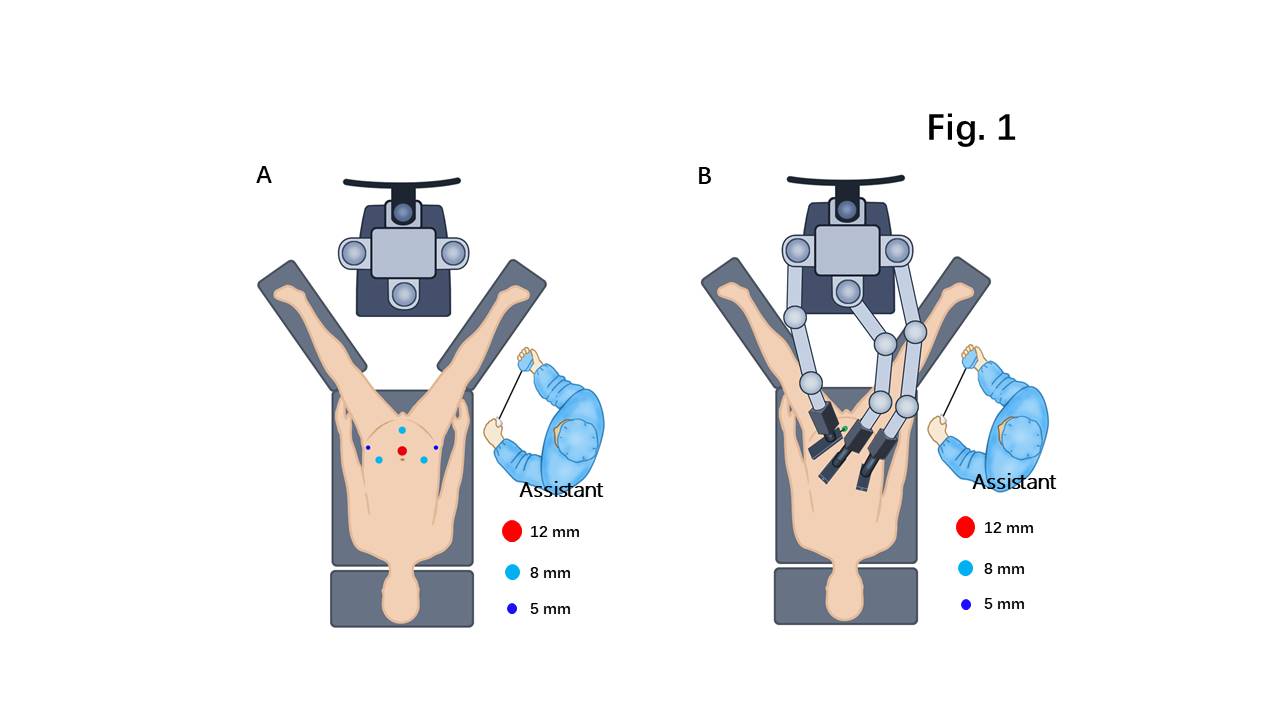
**

**Supplementary Figure. 1** Port site configuration (A), the robot is located between two legs (B). The assistant stands ipsilaterally for each side of RAIL.


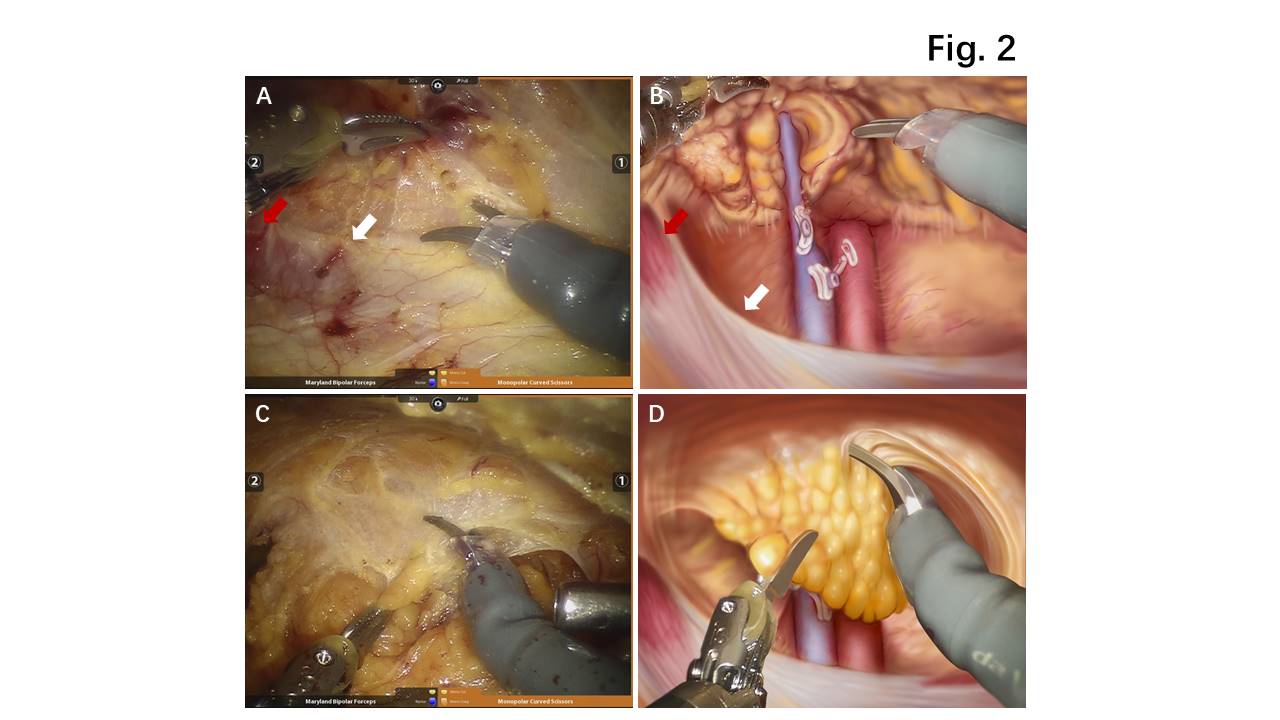


**Supplementary Figure. 2** The deep plane dissection of RAIL (A), its schematic diagram (B) and the superficial plane dissection of RAIL (C), its schematic diagram (D). Red arrow: spermatic cord. White arrow: inguinal ligament.


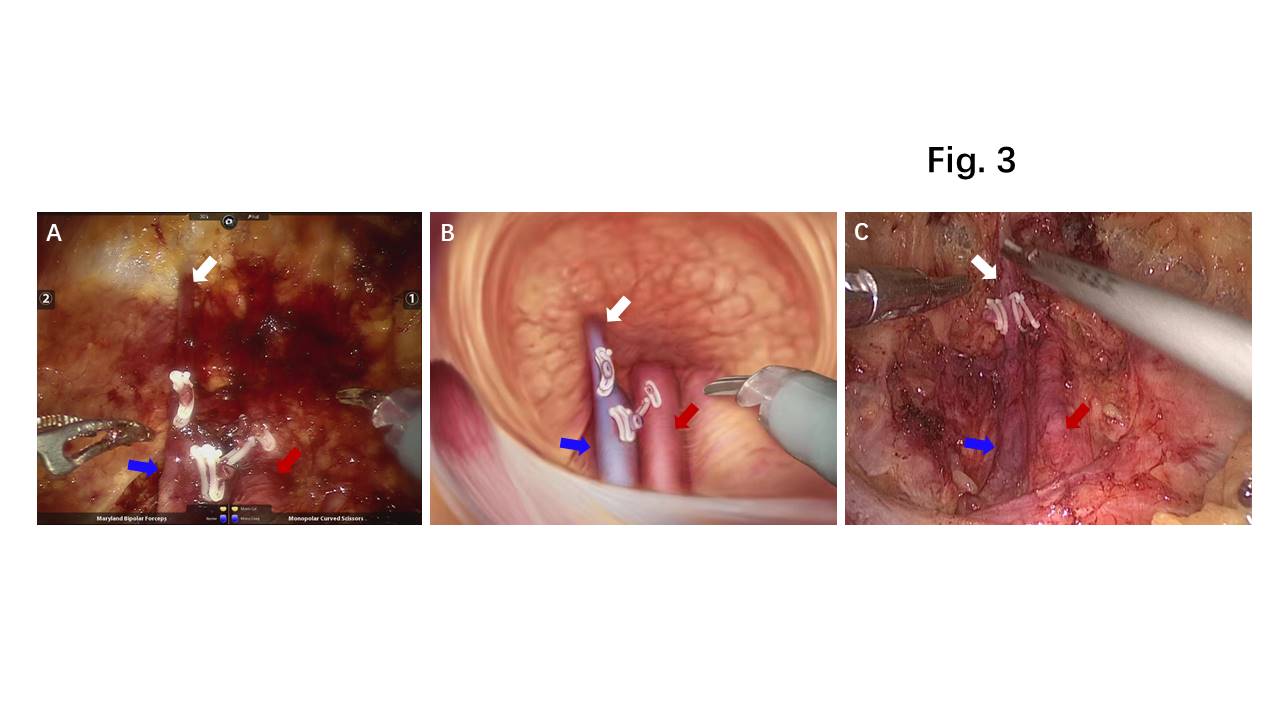


**Supplementary Figure. 3** Intraoperative image of completed field of dissection for RAIL (A), schematic diagram of RAIL(B) and LIL(C). Red arrow: femoral artery. Blue arrow: femoral vein. White arrow: great saphenous vein.
